# ‘Sub-Prime’ Water, Low-Security Entitlements and Policy Challenges in Over-Allocated River Basins: the Case of the Murray–Darling Basin

**DOI:** 10.1007/s00267-020-01303-7

**Published:** 2020-05-20

**Authors:** Harriet Elizabeth Moore, Ian D. Rutherfurd, Murray C. Peel, Avril Horne

**Affiliations:** 1 School of Geography, The University of Lincoln, Lincoln, Lincolnshire UK; 2 School of Geography, The University of Melbourne, Melbourne, VIC Australia; 3 School of Engineering, The University of Melbourne, Melbourne, VIC Australia

**Keywords:** Water policy, Murray–Darling Basin, Water entitlement security, Environmental water, Water market

## Abstract

Environmental policy is often implemented using market instruments. In some cases, including carbon taxing, the links between financial products and the environmental objectives, are transparent. In other cases, including water markets, the links are less transparent. In Australia’s Murray–Darling Basin (MDB), financial water products are known as ‘entitlements’, and are similar to traditional financial products, such as shares. The Australian water market includes ‘Low Security’ entitlements, which are similar to ‘sub-prime’ mortgage bonds because they are unlikely to yield an amount equal to their financial worth. Nearly half the water purchased under the Murray–Darling Basin Plan for environmental purposes is ‘Low Security’. We suggest that the current portfolio of water held by the Australian Government for environmental purposes reflects the mortgage market in the lead-up to the global financial crisis. Banks assumed that the future value of the mortgage market would reflect past trends. Similarly, it is assumed that the future value of water products will reflect past trends, without considering climate change. Historic records of allocations to ‘Low Security’ entitlements in the MDB suggest that, in the context of climate change, the Basin Plan water portfolio may fall short of the target annual average yield of 2075 GL by 511 GL. We recommend adopting finance sector methods including ‘hedging’ ‘Low Security’ entitlements by purchasing an additional 322–2755 GL of ‘Low Security’, or 160–511 GL of ‘High Security’ entitlements. Securing reliable environmental water is a global problem. Finance economics present opportunities for increasing the reliability of environmental flows.

## Highlights

- Water markets and associated products, such as ‘water entitlements’ are often used to reallocate resources for environmental purposes.
- The reliability of water entitlements varies; some are ‘Low Security’ meaning they are unlikely to receive full allocations in most years, while others are ‘high security’ meaning they are more likely to receive full allocations.
- ‘Low Security’ entitlements operate similarly to ‘sub-prime’ mortgage bonds.
- Nearly half of the water entitlements purchased under the Murray–Darling Basin Plan for environmental recovery are ‘Low Security’.
- In the context of climate change, ‘Low Security’ entitlements are likely to yield considerably less than the official Murray–Darling Basin Authority estimates. Thus, the Basin Plan portfolio is likely to fall short of the annual average target of 2075 GL by up to 511 GL.
- Finance economics suggest avenues for improving the reliability of the Basin Plan portfolio, such as ‘hedging’ which would involve purchasing an additional 322–2755 GL of ‘Low Security’, or 160–511 GL of ‘High Security’ entitlements.
- The reliability of water entitlements is as important to the success of environmental management as purchasing the entitlements.
- The low reliability of environmental water entitlements is a global problem. Lessons from the global financial crisis may offer some solutions for ensuring water markets are effective means of improving ecological condition in degraded river basins.

## Introduction

A characteristic of 21st century environmental policy is the use of economic instruments and theories to allocate scarce natural resources (Costanza et al. 1997), such as water (Simpson and Ringskog, 1997), and to address environmental problems, such as climate change (Nordhaus 1994). Some common examples include cap-and-trade policies to reduce water consumption in over-allocated basins (e.g., Thompson et al. 2009), including Australia’s Murray–Darling Basin (MDB) (e.g,. Garrick et al. 2009), pollution taxes (Eskeland and Devarajan 1996; Goulder and Parry, 2008), including carbon taxes (e.g., Nordhaus 1993; Poterba 1991), offsets (Sullivan 2013), including biodiversity offsets (e.g., Coralie et al. 2015), and mitigation banking for wetland (Hallwood 2007; Zedler and Callaway 1999) and stream restoration (Lave 2018; Lave et al. 2018). To be effective, these approaches require transparency, accountability of the parties involved, accounting and monitoring and information such as the amount of water extracted from river basins under a cap-and-trade system, or the number of hectares of wetland necessary to replace the ecosystem services lost as a result of habitat destruction (e.g., Brown and Lant 1999).

The MDB, Australia is an example where the Australian Government implemented a number of economic instruments designed to allocate water more efficiently and sustainably, as well as protecting and restoring the health of the river system. These instruments included a cap and trade system, coupled with the recovery of environmental water through purchases of water rights. In this paper we evaluate an important aspect of the Australian Government’s approach to purchasing water for environmental restoration in the MDB, as articulated in the Murray–Darling Basin Plan (the Plan) (Murray–Darling Basin Authority 2019a, b). We suggest that a key challenge has been translating policy into practice, in this case, appropriate economic mechanisms of water allocation. The central premise of this paper is that the low reliability of nearly half of the water purchased for environmental flows represents a challenge to achieving the environmental objectives of the Basin Plan.

Some economic approaches to resource allocation involve relatively straight forward underlying principles, such as carbon offset trading. While there are many debates about the complexity and geographic implications of carbon offsetting (Bumpus and Liverman 2008), in theory, a license permitting a set amount of carbon emissions can be sold and purchased anywhere in the world because the net outcome remains the same (Lovell and Liverman 2010). One unit of carbon trading is the equivalent of 1 metric tonne of carbon emissions regardless of geographic location, or the source of emissions. Thus, the link between the market product (a license) and the environmental asset (carbon emissions) is transparent^1^.

In other cases, such as mitigation wetland banking (Hallwood 2007; Zedler and Callaway 1999), the link between the market product, and the environmental asset, is much less tangible. This is because the value of traded units varies according to allocated ‘determinations’. For example, under Section 404 of the 1972 Federal Water Pollution Control Act, the US Federal Government permits individuals or companies to destroy natural wetlands on the condition that they compensate the loss by creating an equal amount of wetland of comparable quality (Robertson 2004). A company may destroy 100 hectares of high-quality wetland, and compensate by creating 200 hectares of 50% ecologically functional wetland. Modelling involving complex algorithms is used to establish the quality of the wetland, and thus the allocated ‘determination’ of the asset; if the wetland is 50% functional, the determination is 0.5% per hectare (Robertson 2004). Thus, the party responsible must create twice as much mitigation wetland as the amount lost.

This approach to mitigation wetland banking has been heavily criticised for a number of reasons, including the fact that the ecological modelling involves substantial uncertainties (e.g., Brown and Lant 1999; Burgin 2008; Robertson 2004). Hectares of wetland of variable quality are pooled together under the assumption that a certain amount of low-quality wetland will result in the equivalent biophysical function of a target amount of high-quality wetland. Furthermore, it is assumed that modelling can be used to identify the ratio of how many low-quality hectares equal one high-quality hectare. To be effective, a large enough amount of low-quality wetland must be established to ‘hedge’ the risk associated with low ecological functionality.

A similar logic to mitigation wetland banking underlies the Australian Government’s approach to reclaiming water for environmental purposes in the MDB. In both cases, large amounts of low-quality assets are assumed to be equal to a smaller amount of high-quality assets. In this paper we present an analysis of the MDB environmental water recovery, explore the implications of using ‘High’ and ‘Low’ security water entitlements for future environmental water security, and provide key recommendations based on lessons from the financial sector. In the following we present:

- An overview of the approaches used to provide environmental water in the MDB;
- A brief history of the institutional origins of different security water classes;
- An analysis of all available ‘Low Security’ entitlement allocation records in the MDB (between 2002 and 2018), including calculation of likely future yields, particularly in the context of climate change; and
- Some recommendations for increasing the security of environmental water.

## Major Water Reform in the MDB

**Fig. 1:**
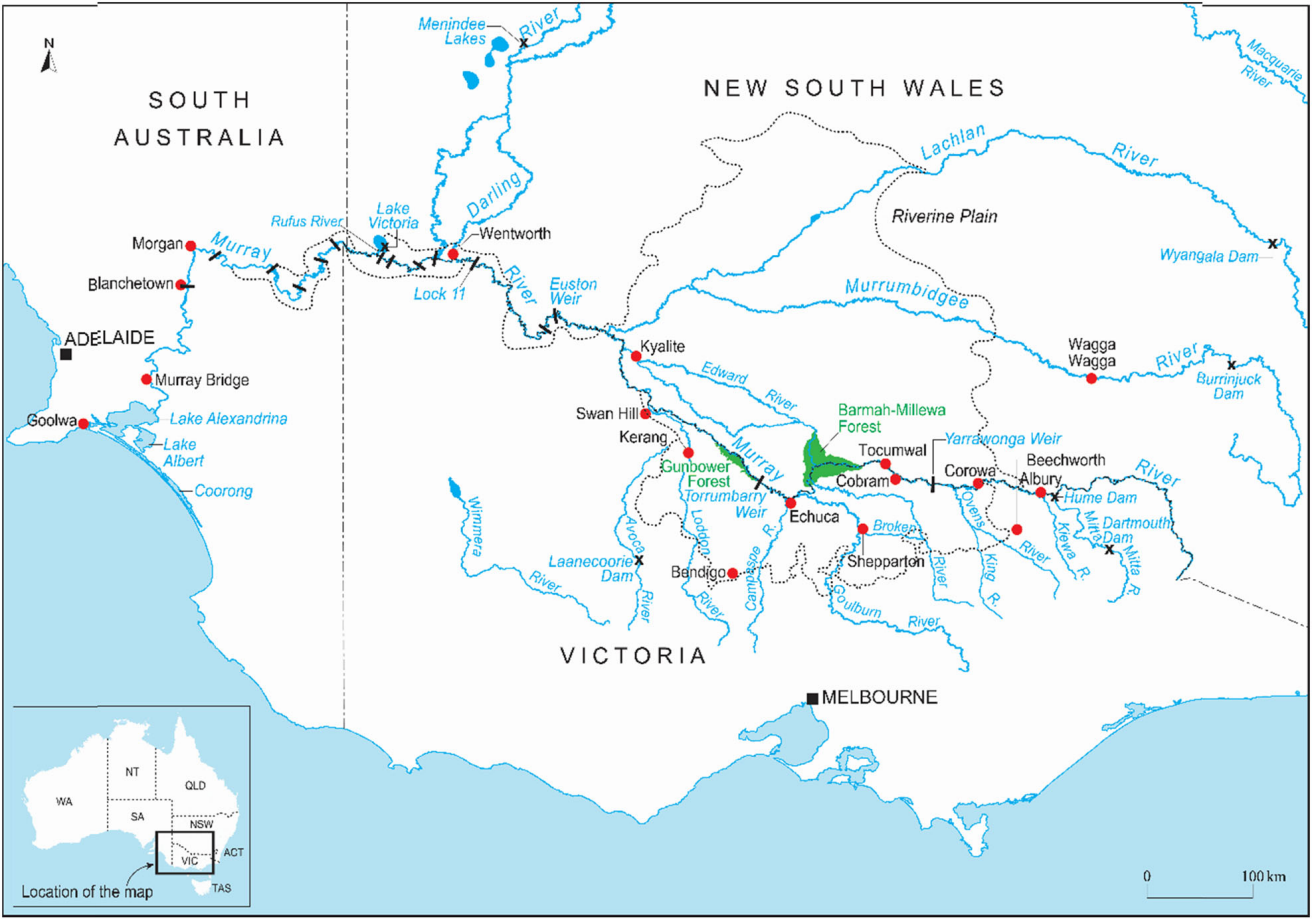
Map of the southern Murray–Darling Basin, Australia

The MDB is located in south-eastern Australia, and contains the nation’s longest river system, including the Murray and Darling rivers, and their tributaries (Fig. 1). The Basin covers approximately 1 million sq km and runs through the States of South Australia, New South Wales, Victoria, Queensland and the Australian Capital Territory. Like many major river systems globally, the MDB has a history of extensive development, large scale irrigation infrastructure and increasing volumes of water extraction. Agricultural production in the Basin consumes ~60% of agricultural water used in the nation, and generates nearly 40% of all income from the agricultural sector in Australia (Leblanc et al. 2012). Over the past 100 years, the ecology, hydrology and geomorphology of the MDB system has been fundamentally altered by impoundment, diversions, extraction, vegetation clearing and the introduction of non-native species of flora and fauna (e.g., Pittock and Finlayson 2011; Walker 1985; Walker and Thoms, 1993). As a consequence, the system has seen large scale environmental impacts with public and policy interest sparked by a number of large algal blooms throughout 1991–1992 (Guest 2017).

The federal government took control of a number of aspects of water management triggered by the destruction of internationally recognised Ramsar wetlands (e.g., Kingsford 2000). Previously, natural resource management was the jurisdiction of individual states. Following major environmental problems, such as salinization and persistent drought, the management of the MDB has increasingly been overseen by federal institutions, such as the Murray–Darling Basin Commission, and later, the Murray–Darling Basin Authority (MDBA). The Living Murray project (TLM), established in 2002, was one of the first collaborative environmental programmes initiated by the Murray–Darling Basin Ministerial Council under the Murray–Darling Basin Commission. With ongoing deterioration of the river system and mounting public concern, the Water Act 2007 further cemented the federal government’s role in managing the basin.

Under the Water Act 2007, the Government established a federal agency, the MDBA who were responsible for developing the Murray–Darling Basin Plan (the Basin Plan). The two key mechanisms in the Basin Plan aimed at addressing the degraded condition of the MDB are the setting of a limit to the amount of water permitted for extraction, known as the sustainable diversion limit (SDL), and recovering water for environmental restoration, known as ‘environmental water’. A second federal organisation, the Commonwealth Environmental Water Holder (CEWH^2^), was established under the Water Act 2007 to manage environmental water.

In 2012, the MDBA released the Basin Plan, stipulating a recovery target of 2750 gigalitres (GL) of water (Murray–Darling Basin Authority 2019a, b). In 2018 this target was revised downwards to 2075 GL (Murray–Darling Basin Authority 2019a, b). While there has been much debate over the recovery target (e.g., Cosier et al. 2012), the legitimacy of the recovery target is not the focus of this paper. We focus instead on how water recovery was achieved. The two main mechanisms for water recovery were through the water market by purchasing water ‘products’ known as entitlements, and by reducing the volume of water extracted by the irrigation sector by funding irrigation efficiency projects, and returning water savings to the Commonwealth for environmental watering. The objectives of recovering water through these programmes were ‘multiple, poorly defined and at times conflicting’ (Horne et al. 2011, pg. 380), but included helping ease the transition to the lower levels of water available to consumptive users as a result of the SDL. The methods used to recover water were based on both economic efficiency and political palatability (Horne et al. 2011). There has been criticism of the efficiency of the water recovery methods adopted by the Australian Government, including the negative economic impacts of recovering water through irrigation efficiency upgrades rather than through purchasing directly through the water market (e.g., Grafton and Horne, 2014; Qureshi et al. 2011). While the focus of the recovery programme was on the most efficient and politically palatable way to recover water, the recovery target is a surrogate for how much water is required to achieve particular environmental outcomes.

The Basin Plan and water recovery targets do not explicitly consider the implications of climate change. However, the Basin Plan is an adaptive process with reviews at regular intervals, and the consideration of climate change is recognised as an essential improvement for the next version in 2025. The analysis in this paper highlights the importance of considering the type of product, (known as ‘entitlements’) and the mechanisms that are used to provide environmental water and how they will perform under climate change. This type of analysis will be important for informing the next round of policy settings.

## The Security of Water Entitlements for Environmental Flows in the MDB

The MDB has a complex set of formal water allocation mechanisms. While the States retain ownership of water, individual water users can hold a water right that gives them legal entitlement to an annual allocation (similar to a dividend) of water based on how much water is in storage across the system at a particular time. The likely allocation (or annual return) varies across individual rivers depending on local climate, but there are also often a range of water products within each system. Some entitlements are ‘High Security’ while others are ‘Low Security’ (Ancev 2015). The ‘security’ of water entitlements refers to the likelihood that the full amount of water will be allocated to an entitlement in any given year. The introduction of ‘High Security’ and ‘Low Security’ entitlements for water allocation in the MDB followed the establishment of a cap-and-trade policy (the Cap) by the Federal Government. The MDB diversions cap, limiting diversions to 1994 levels (Quiggin 2001) was introduced in 1995 under the River Murray Waters Agreement to address the over-allocation of water in the system. Over-allocation meant that entitlement holders were unable to receive full allocations. Thus, a net reduction was required in the allocation of water to existing entitlement holders. This could have been achieved by revoking a proportion of existing entitlements. However, the preferred approach was to implement a hierarchy of entitlements. Existing entitlements were given a ‘rating’ to indicate priority; in times of low rainfall and river flow, water is allocated to high-priority entitlement-holders first, and any remaining water is distributed between low-priority entitlement-holders after that (Quiggin 2001). The terminology adopted to describe low-priority entitlements varied across the States of the MDB, called medium security in Queensland, General Security in NSW and Low Security in Victoria. However, these categories are equivalent to each other in terms of likelihood of receiving full allocations. Thus, for the purposes of this paper, we refer to all low-priority entitlements as ‘Low Security’, and high-priority entitlements as ‘High Security’^3^.

‘Low Security’ entitlements were established on the premise that those entitlements were unlikely to receive full allocations in most years; only a proportion of the total amount would be available for allocation to these entitlements. At the beginning of the agricultural season, and throughout the year, water authorities announce ‘determinations’ for water entitlements in each sub-basin in the MDB. Similar to the case of mitigation wetlands, water entitlement determinants specify the ‘quality’ of the asset, in this case, the amount of water per megalitre (ML) of an entitlement that is available for use. Thus, an allocation of 100 ML to a 100 ML entitlement would be announced as a determinant of ‘1’, while an allocation of 50 ML to the same entitlement would be announced as ‘0.5’. A ‘High Security’ entitlement for 100 ML is likely to be allocated close to, if not exactly, 100 ML of water for use in a given year. A ‘Low Security’ entitlement is likely to receive a much smaller proportion in any given year compared with ‘High Security’ entitlements because ‘High Security’ entitlements are allocated first, and any remaining water in storage is divided between all other holdings.

Under the Basin Plan a portfolio of water entitlements has been acquired for environmental watering, including both ‘High Security’ and ‘Low Security’ entitlements. The security of the water purchased by the CEWH under the Basin Plan varies considerably. As of November 11th, 2019, the CEWH holds 2755 GL of registered water entitlements (excluding ground water entitlements) for environmental purposes (Australian Government Department of the Environment and Energy 2019).

In theory, purchasing a large amount of ‘Low Security’ entitlements should yield a reliable amount of water equivalent to a smaller holding of ‘High Security’ entitlements, particularly if the ‘Low Security’ entitlements are purchased from a range of river basins. At any given time, some entitlements should receive allocations. This approach is a form of ‘hedging’ risk; the risk that any one ‘Low Security’ entitlement will fail to produce water for environmental flows is ‘hedged’ by purchasing many of these entitlements in a number of river basins. Hedging the risk of ‘Low Security’ water underpinned how the Australian Government procured water for TLM Project. The TLM involved acquiring entitlements to provide a long-term annual average yield (LTAAY) of 500 GL for environmental watering at six ‘icon’ sites of high ecological, recreational, cultural and heritage value. To achieve a LTAAY of 500 GL, the MDBA purchased ~975 GL worth of water entitlements of varying security, including 870 GL of ‘Low Security’ entitlements (570 GL of NSW ‘General Security’ and ‘Supplementary’, and 300 GL of Victorian ‘Low Security’ entitlements), and 104 GL of ‘High Security’ entitlements (2 GL from NSW, 58 GL from Victoria, and 44 GL from South Australia) (Murray–Darling Basin Authority 2011). Overall, of the target LTAAY of 500 GL, 104 GL was obtained by purchasing ‘High Security’ entitlements, while the remaining 396 GL LTAAY was secured by purchasing more than double this amount in ‘Low Security’ entitlements. These figures were determined by modelling which suggests that, based on 100 years of historical river flow data, on average ‘Low Security’ entitlements are likely to yield 0.5/ML, or half the amount of water compared with the value, in MLs, of the entitlement.

Concerns about the reliability of these ‘Low Security’ entitlements, and the modelling and methods used to determine long-term yields, were raised early in the planning phase of TLM Project (e.g., Cox et al. 2006). In some cases, those concerns have been realised. For example, Horne (2017) observes that, ‘*Some [Living Murray] entitlements, specifically the Victorian Goulburn Valley and Murray Valley low reliability entitlements, have received a total allocation of only 5GL since becoming part of the portfolio*’. (p.1005). However, in theory, purchasing a large amount of ‘Low Security’ entitlements should ‘hedge’ the risk, and produce a long-term average close to 500 GL.

Importantly, the modelling used to determine the LTAAY for both TLM and Commonwealth environmental water holdings is based on historic climate, rainfall and stream flow data, irrigation use, and dam storage. The MDBA Hydrologic Modelling Report (Murray–Darling Basin Authority 2012) specifies that long-term average water availability scenarios related to reclaiming environmental water were modelled using climate data covering the period of 1895–2009. The modelling method to determine LTAAY did not take into account climate change scenarios (Murray–Darling Basin Authority 2012). Climate change projections of rainfall indicate that the region of the MDB may experience drier conditions into the future, such as the conditions experienced during the recent 15-year period of low rainfall (Dey et al. 2019). In this scenario, the amount of water allocated to ‘Low Security’ entitlements over the past 15 years may be more indicative of future yields compared with MDBA estimations based on average climatic conditions over the past 100 years (Australian Government Department of the Environment and Energy 2019). Thus, if future rainfall is similar to the past 100 years, estimates of yields of water per ML from ‘Low Security’ entitlements are likely to be accurate. However, in drier conditions, such as the most recent 15 years, these estimates may be considerably higher than actual available allocations, and the amount of water allocated to the entitlements held in the CEWH portfolio may fall short of the target 2075 GL. These concerns may also apply to the TLM water entitlement portfolio.

In the following sections we investigate the amount of water per ML allocated to ‘Low-Security’ entitlements in the past 10–15 years in the MDB, and consider the implications for ‘Low Security’ allocations in the context of climate change. We acknowledge that the past 10–15 years may not be entirely indicative of future climatic conditions. Our analysis is intended to demonstrate how the CEWH portfolio may function in drier conditions.

## Methods and Results

Our analysis of ‘Low Security’ entitlement allocations draws on publically available data about the yearly ‘determinants’ allocated to those entitlements in the states of the MDB, specifically Queensland, New South Wales and Victoria. The data period ranges from 2002 and 2018. We used two approaches to consider whether ‘Low Security’ water entitlements purchased under the Basin Plan are likely to yield an annual average amount of water close to the target amount of 2075 GL. Firstly, we examined the amount of ‘Low Security’ entitlements held under the Basin Plan and established whether those entitlements were ‘hedged’ against the risk of low yields. Secondly, we examined the history of allocation determinations, and calculated the likely proportion of allocations to those entitlements in the context of future climate scenarios, including the scenario in which future climate reflects the past 100 years, and the scenario in which future climate is similar to the past 15 years of low rainfall. The following summarises the methods used to conduct these analyses, and the results of each approach.

### Proportion of ‘Low Security’ Entitlements Held under the Basin Plan

To identify the proportion of ‘Low Security’ entitlements purchased under the Basin Plan, and to identify whether these entitlements were ‘hedged’, we examined the register of water entitlements held by the CEWH (Australian Government Department of the Environment and Energy 2019). The approach taken for obtaining 500 GL LTAAY for TLM project suggests that, in climatic conditions similar to that of the past 100 years, ‘Low Security’ entitlements should yield 0.5/ML, or half the amount of water compared with the amount of purchased entitlements. Thus, we assume that double the amount of ‘Low Security’ entitlements is required to ‘hedge’ risk in the context of the CEWH portfolio. It is important to note that the current portfolio does not represent full recovery; recovery is ongoing. We consider the amount of additional water required to meet the long-term average target of 2075 GL, and whether the proportion of ‘Low Security’ entitlements is sufficient to meet the target in the context of future climate change.

Table 1 summarises data obtained from the CEWH register (Australian Government Department of the Environment and Energy, 2019), including the amount of ‘High Security’ and ‘Low Security’ water entitlements held under the Basin Plan, and the expected LTAAY, calculated using modelling methods established by the MDBA. Expected yield refers to the amount of water that the modelling suggests will be allocated to an entitlement, as a proportion of 1 ML. Both ‘Low Security’ and ‘Other’ entitlements are anticipated to yield approximately half the amount of water as that held under entitlement^4^. These estimates are consistent with the modelling used for TLM project which indicated that a 1 ML ‘Low Security’ entitlement should yield approximately half a ML of water annually (0.5/ML). However, the register indicates that the expected yield of the total portfolio held under the Basin Plan (including both ‘High’ and ‘Low’ security entitlements) is only 1913 GL, which is 162 GL below the 2075 GL target. Thus, at the current time, the Basin Plan portfolio is only partially ‘hedged’ against the risk of low-yielding entitlements. Water recovery for the Basin Plan portfolio is ongoing. Given that ‘Low Security’ entitlements are expected to yield approximately half the registered amount, an additional 324 GL of these entitlements would be required to achieve the long-term annual average target.

**Table 1:** Summary of High, Low, and Other^a^ entitlements held by the CEWH under the Basin Plan, including expected yields based on MDBA modelling

Security type	Amount (ML)	Expected yield
High	892,370	837,353
Low	**1,092,440**	**632,977**
Other	819,761	465,598
Total	2,804,571	1,935,928

^a^‘Other’ holdings refer to the combined total of holdings of varying security that pertain to specific regions only. ‘Other’ Queensland holdings include ‘Un-supplemented’, and ‘Overland’. ‘Other’ New South Wales holdings include ‘Conveyance’, ‘Supplemented’, and ‘Unregistered’. ‘Other’ Victorian holdings include ‘Bulk’ entitlements

### Past Allocations and the Expected Yield of ‘Low Security’ Entitlements in the Context of Climate Change^5^

The modelling used to determine LTAAY is based on long-term average rainfall data. However, by 2050 climate in the MDB may be more similar to the past 15 years of low rainfall and river flow (Dey et al. 2019). Thus, our second approach was to obtain records of seasonal allocations to ‘Low Security’ or equivalent entitlements in NSW (New South Wales Department of Industry 2019; Water NSW 2019), Victoria (Victoria State Government Department of Land Water and Planning 2019) and Queensland (Personal Communication, Sunwater Queensland, 12 March 2019^6^). This data was used to estimate the average historic allocation to ‘Low Security’ entitlements in sub-basins of the MDB. These average values were used to calculate the amount of water that is likely to be allocated to ‘Low Security’ entitlements held under the Basin Plan in the event that climatic conditions are similar to the past 15 years.

While there is evidence to suggest future climatic conditions may be similar to the past 15 years (Dey et al. 2019), we acknowledge that the extreme conditions characteristic of the Millennium Drought (1996–2010) may impact our analysis (e.g., Crase et al. 2012). Thus, we also investigated whether there was a difference in the amount of water allocated to ‘Low Security’ entitlements during the drought, compared with following the drought in 2010.

An alternative approach could be to identify all periods of drought and compare allocations during these periods to all periods when drought has not occurred. Since 2010 there have been multiple periods of drought, such as 2014–2015 when total inflows into the Murray River fell to 60% compared with the long-term medium (Murray-Darling Basin Authority, 2015). However, drought conditions can occur for multiple years before reducing dam storage to the point of severely impacting entitlement allocations (Van Loon 2015). This is because the storage dams in the south eastern MDB have a capacity of several times annual average inflows, allowing for carryover of water stored from a previous year for allocation and use in later years. Thus, several years of meteorological drought (low precipitation) can occur without severely reducing storage levels, and thus, without impacting entitlement allocations (Craik and Cleaver, 2008). For example, in the early phase of the Millennium Drought in the sub-basins of Victoria in the MDB (1996–2005) allocations to ‘Low Security’ entitlements dropped from 80 to 54%. By comparison, the average annual allocation to ‘Low Security’ entitlements in sub-basins in Victoria between 2007 and 2019 ranged between 23% in the Campaspe and 0% in the Goulburn, Loddon and Murray rivers. Therefore, our analysis compares allocations to ‘Low Security’ entitlements during the Millennium Drought to allocations after the cessation of the drought because long periods of drought have a greater impact on allocation determinants than short periods of drought.

Complete records of seasonal allocations to ‘Low Security’ entitlements as a proportion of 1 ML were obtained for nine basins in NSW, three basins in Queensland, and five basins in Victoria (Table 3). The range of years for which allocation data was available varied between States, as demonstrated in Table 2, and also annually in single States. For example, in NSW, records from 2015 include allocation values for the months April to December, while records from 2016 include values for the months January to December.

**Table 2:** Basins in NSW, Queensland and Victoria for which ‘Low Security’ allocation data were obtained^7^

State	Basins	Years
NSW	Gwydir, Lachlan, Lower Darling, Macquarie-Castlereagh, Murrumbidgee, Naomi, NSW Boarder Rivers, NSW Murray and Peel	2004–2018
QLD	Condamine and Balonne, QLD Border Rivers and Warrego–Paroo–Bulloo–Nebine	2002–2018
VIC	Broken, Campaspe, Goulburn, Loddon and Murray	2007–2019

Increasing allocation announcements are made throughout the irrigation season as new inflows enter storage, often with multiple allocation increases announced in a single month. For this reason the highest value for each month was taken as the final monthly allocation.

In total, we analysed 690 monthly announcements about allocations to ‘Low Security’ water entitlements over the years 2002–2018 from NSW (*N* = 412), Victoria (*N* = 72) and Queensland (*N* = 205). Using this data we calculated the long-term average allocation of ‘Low Security’ entitlements to each individual basin as a proportion (0–1). Table 3 summarises the number of ‘Low Security’ entitlements held by the CEWH in each basin (ML), the long-term average yield of those entitlements based on MDBA modelling (ML), as well as the long-term allocation based on our analysis of actual allocation data, the average amount of water those allocations would yield, and the difference between MDBA-modelled yields and our calculations.

**Table 3:** The number of ‘Low Security’ entitlements held by the CEWH in QLD, NSW and VIC basins, including the average yield predicted on the basis of MDBA modelling, the average allocation calculated using available allocation data between 2002 and 2018, average yield based on allocation data, and the difference in expected yield between MDBA modelling and predictions based on available data

State	Basin	Entitlements held by CEWH (ML)	Long-term average yield based on MDBA modelling (ML)	Long-term average allocation based on actual data (as % of ML)	Long-term average yield based on actual data (ML)	Difference between MDBA-modelled yield and yield based on actual data (ML)
QLD	Border rivers	15,540	5241	0.71	11,009.85	+5768.85
	St. George	45	43	0.70	31.53	−11.47
NSW	Border rivers	2806	946	0.40	1134.11	+188.09
	Gwydir	86,923	34,020	0.12	10,589.59	−23,430.41
	Lachlan	86,923	34,422	0.14	12,096.78	−22,325.22
	Lower Darling	21,564	20,076	0.45	9,605.78	−10,470.22
	Macquarie/Cudgegong	126,224	65,132	0.11	13,649.8	−51,482.2
	Murray	369,629	258,371	0.09	34,566.09	−223,804.9
	Murrumbidgee	286,467	169,302	0.09	25,923.5	−143,378.5
	Nanoi (upper and lower)	13,653	10,281	0.23	3,171.73	−7109.27
	Peel	1257	263	0.29	367.49	+104.49
VIC	Broken	4	3	0.38	1.52	−1.48
	Campaspe	395	194	0.23	89.2	−104.8
	Goulburn	42,467	19,265	0	0	−19,265
	Loddon	527	142	0	0	−142
	Murray	35,413	15,276	0	0	−15,276
	Total	1,074,297	632,977	NA	122,237	−510,740

The data presented in Table 3 were used to calculate the future yield of all entitlements reported in the CEWH register associated with the Basin Plan target of 2075 GL. We multiplied the average allocation proportion by the amount of ML of ‘Low Security’ water held in each basin to determine the likely yield of those entitlements under climate conditions similar to the past 15 years. For example, the long-term average allocation of water to ‘Low Security’ entitlements in the Murray Basin of NSW is 0.09/ML, and the amount of water held under those entitlements is 369,629 ML. The result of multiplying these values is 34,566.09 ML, indicating a 223,804.9 ML shortfall between the long-term average annual yield estimated on the basis of MDBA modelling for the NSW Murray, and the likely yield based on recent allocations.

These figures were then used to determine the difference between official predictions of future yield from all entitlements held in the CEWH compared with our calculations based on historic allocations to ‘Low Security’ entitlements. Table 4 shows the amount of entitlements of ‘Low’, ‘High’ and ‘Other’ security water held in NSW, Victoria and Queensland under the Basin Plan, expected LTAAY yield based on MDBA estimates, as well as expected yield based on our calculations of average annual allocation to ‘Low Security’ entitlements over the past 10–15 years, as a proportion of 1 ML.

**Table 4:** Summary of: amount of ‘High’, ‘Low’, and ‘Other’ entitlement holdings in the Murray–Darling Basin held by the Commonwealth Environmental Water Holder (CEWH), expected long-term annual average yield (LTAAY), long-term average percentage (%) of allocation per Megalitre (ML) in Queensland, New South Wales and Victoria based on past allocation data, expected average annual yield based on past allocation data, and difference between expected LTAAY and expected yields. South Australia ‘High Security’ included, and groundwater entitlements excluded for accuracy

Region	Water product (Entitlement security)	Entitlement holdings registered with CEWH (ML)	Long-term average annual yield (ML)		
			Modelled (based on MDBA modelling^a^)	Actual (based on entitlement allocation data from 2002 to 2018)	Difference between modelled and actual long-term annual yield (ML)
QLD	High	0	0		
	**Low**	**15,585**	**5284**	**11,041**	**+5757**
	Other	237,992	121,911		
	Total	253,577	127,195	**132,952**	
	% Expected yield		50	**52**	
NSW	High	40,555	36,561		
	**Low**	**998,049**	**592,810**	**111,105**	**−481,705**
	Other	553,769	298,551		
	Total	1,592,372	927,922	**446,217**	
	% Expected yield		58	**28**	
VIC	High	690,398	655,517		
	**Low**	**78,807**	**34,880**	**91**	**−34,789**
	Other	28,000	22,568		
	Total	797,205	712,965	**678,176**	
	% Expected yield		89	**85**	
SA	High	161,417	145,276		
	–	–	–		
	–	–	–		
Whole Murray–Darling Basin	High	892,370	837,353		
	**Low**	**1,092,440**	**623,977**	**122,237**	**−510,737**
	Other	819,761	443,030		
	Total	2,804,571	1,913,360	**1,402,620**	
	% Expected yield		68	**50**	
	**Total after removing the shortfall**			**1,402,620**	
	**% Expected yield after removing the shortfall**			**50.01**	

^a^Estimates sourced from CEWH register, September, 2019

Overall, 1,092,440 ML of ‘Low Security’ water entitlements are held under the Basin Plan, combining entitlements from NSW, Victoria and Queensland. The MDBA calculates a LTAAY of 623, 977 ML using modelling that suggests these ‘Low Security’ entitlements should yield ~0.5/ML. Based on historic allocations, over the last 15 years, to ‘Low Security’ entitlements, we calculate an average annual yield of only 122, 237 ML, and thus a shortfall of 510, 737 ML compared with MDBA estimates. On average, across all basins, ‘Low Security’ entitlements yield only 0.12/ML in Victoria, 0.21/ML in NSW and 0.7/ML in QLD. Overall, in climatic conditions similar to the past 15 years, we estimate an average annual yield of 1,402,620 ML from the 2,804,571 ML of entitlements held under the Basin Plan. Thus, if future climatic conditions in the MDB reflect the last 15 years, it is possible that the CEWH portfolio may yield as little as 50% of the total entitlement, compared with 68% based on the long-term historical average.

**Fig. 2:**
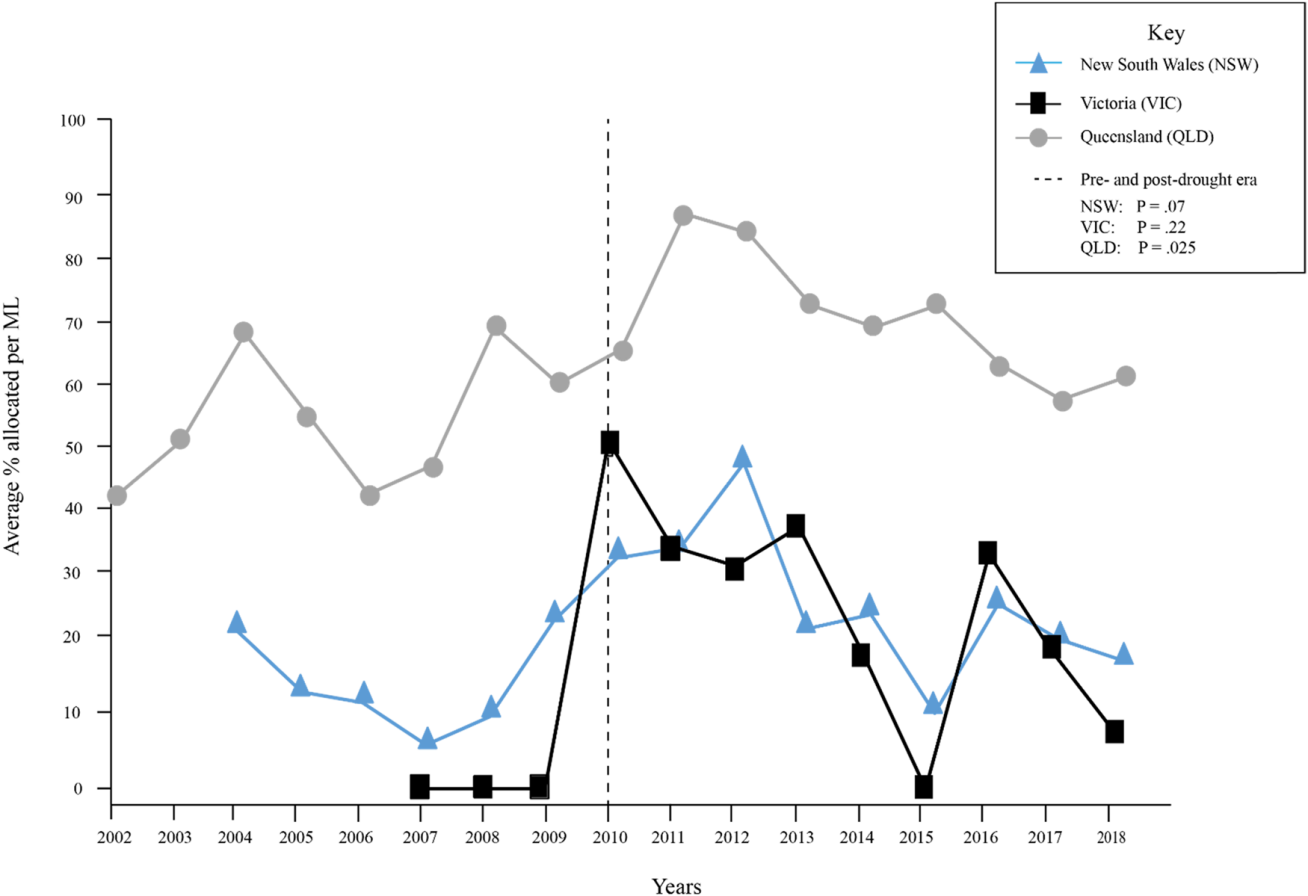
The average annual percentage per Megalitre (ML) of ‘Medium security’ entitlements allocated in Queensland between 2002 and 2018, ‘General security’ entitlements allocated in New South Wales between 2004 and 2018, and ‘Low security’ entitlements allocated in Victoria between 2007 and 2018. The dotted line represents the end of the Millennium Drought. *P* values in the Key demonstrate that the amount of water allocated to ‘General’ security entitlements in New South Wales, and ‘Low’ security entitlements in Victoria, as a proportion of 1 ML, was not significantly different in years following the drought, compared with drought years. The amount of ‘Medium security’ entitlements allocated in Queensland was greater after the drought, compared with drought years

The annual average allocations to ‘Low Security’ entitlements in NSW, Queensland and Victoria, as a proportion of 1 ML, are presented in Fig. 2. Over the period for which records are available, ‘Low Security’ entitlements in Queensland received the highest allocations. By comparison, allocations in Victoria and NSW were lower, and similar.

Three *t*-tests were computed to test the difference in mean water (*M*) allocated to ‘Low Security’ entitlements after the cessation of the Millennium Drought compared with during drought years. The results indicate that there was no difference between the amount of water allocated to ‘Low Security’ entitlements in river basins in NSW after (*M* = 0.19, *SD* = 0.29) and during (*M* = 0.14, *SD* = 0.23) the drought, *t*(410) = −1.7, *P* = 0.076. Similarly, there was no difference between the amount of water allocated to ‘Low Security’ entitlements in river basins in Victoria after (*M* = 0.23, *SD* = 0.34) and during (*M* = 0.13, *SD* = 0.34) the drought, *t*(70) = −1.24, *P* = 0.22. In contrast, analysis revealed that more water was allocated to ‘Low Security’ entitlements in river basins in Queensland after the end of the drought (*M* = 0.7, *SD* = 0.36), compared with during the drought (*M* = 0.6, *SD* = 0.36), *t*(204) = −2.23, *P* = 0.027. The *P* values for these three analyses are displayed in Fig. 2.

## Discussion

The establishment of water entitlements and water markets is a common response to scarcity. This approach has been adopted to reallocate water resources between competing users in the United States of America (e.g., Marston and Cai 2016), China (e.g., Wang et al. 2018) and elsewhere (Endo et al. 2018). To our knowledge, the determination of ‘Low’ and ‘High’ security entitlements is unique to the Australian water market. However, similar economic instruments operate elsewhere and present similar challenges for water management. In the Limarí River and Diguillín River basins of Chilé, the water market has been used to transfer irrigation rights to achieve minimum sustainable flows (Kretschmer et al. 2017). However, allocations of water to rights obtained for instream flows usually fall below the legally permissible value of the rights, resulting in substantial shortages. Similarly, in America ‘senior’ water rights established earlier in history out-rank the reliability of more recently established ‘junior’ rights, and environmental flow portfolios include both products. Much like the case of the MDB, portfolios of water products of variable reliability often fail to produce target yields. For example, in the drought year of 2015, actual available stream flow to the Whychus Creek was <60% of the baseline target, despite the fact that 90% of the target water rights had been secured for environmental watering (Kendy et al. 2018). The shortfall indicates the low reliability of the water rights portfolio. Thus, the reliability of environmental water rights is as critical to the success of environmental management as the actual acquiring of water rights, particularly in drought affected regions. The challenges and implications presented in our analysis also apply to any resource management scheme that involves bundling ecological assets of variable quality together to achieve a target amount of high-quality ecosystem, such as biodiversity offsetting (Apostolopoulou and Adams 2017) and mitigation wetland banking (e.g., Hallwood 2007).

### ‘Sub-Prime’ Water

Under the Basin Plan, the target for water recovery is to hold enough water entitlements to produce an annual average yield of 2075 GL. To achieve this goal, to date a portfolio of more than 2800 GL of entitlements has been acquired (with ongoing efforts to recover additional volumes of water), including both ‘High Security’ and ‘Low Security’ entitlements. The underlying assumption is that on average, ‘Low Security’ entitlements should produce a reliable yield equivalent to approximately half the amount of ‘High Security’ entitlements. Thus, 1 GL of water can be obtained by purchasing 2 GL worth of ‘Low Security’ entitlements. We suggest that this approach to implementing the Basin Plan is analogous to the ‘sub-prime’ mortgage market in the lead-up to the global financial crisis (GFC). This comparison is not intended to diminish the validity of Australian water policy. Rather, the lessons learnt by the finance sector after the GFC offer some options for improving the reliability of environmental water, and increasing public confidence in the Basin Plan.

In this analogy, the methods used to recover water are comparable to the creation of financial products, known as collateralised debt obligations (CDOs), by finance firms in the years preceding the GFC. A CDO is a pooled collection of cash-flow generating assets, such as mortgages, of varying degrees of security (Longstaff and Rajan 2008). These assets include BBB-rated mortgages, defined as mortgages with a high risk of payment default and AAA-rated mortgages, defined as those mortgages with a high probability of reliable payment. A CDO, comprising both BBB and AAA mortgages, is divided into shares and sold on the basis that the risk associated with low security assets is distributed evenly between investors. Prior to the GFC, firms created CDOs from low-yielding BBB-rated mortgages (Fender et al. 2008). As these sub-prime mortgages began to fail, the cash-flow yield of CDOs fell dangerously below the expected return.

There are two points of comparison that offer important insights into the future sustainability of the CEWH portfolio. Firstly, much like the content of CDOs, the security of the water purchased by the CEWH under the Basin Plan varies considerably. Similar to mortgages, some entitlements are ‘High Security’ (equivalent to AAA-rated mortgages), while others are ‘Low Security’ (equivalent to BBB-rated mortgages) (Ancev 2015). Nearly 40% of entitlements held by the CEWH are low-yielding assets. Thus, we suggest that, like the content of CDOs, the portfolio is ‘sub-prime’, and poses considerable risk to achieving target environmental flows in the MDB. Secondly, in the case of the finance sector, ‘*the failure of markets to recognise systemic disequilibria because of cognitive bias*’ (Ülgen 2013, p. 498) played a large role in destabilising the mortgage market. In this context, cognitive bias refers to the tendency for cohesive groups, such as policy makers, to reinforce their own beliefs by selecting information that concurs with desirable world-views, while excluding alternative perspectives, and associated information, from decision-making forums (Palley 2009). Bias of this nature is the subconscious outcome of institutional norms within complex economic systems, and transcends the intention, purpose and responsibility of any individual, government or organisation. Thus, the financial crisis of 2008 was due to the failure of institutions to question and challenge dominant paradigms, such as that future trends in the house market would reflect past trends (Ülgen 2013). In the following we examine some of the characteristics of the Australian water market, suggest that similar ‘systemic disequilibria’ threatens the future availability of environmental water in the MDB, and offer some suggestions for improving the reliability of environmental water.

### Risks to the Future Availability of Environmental Water

An assumption of the current approach to securing environmental water in Australia is that the estimated yields will reflect the future availability of water. Importantly, ‘Low Security’ entitlements purchased from basins in New South Wales account for nearly 40% of the total registered entitlements held by the CEWH for the Basin Plan (Table 3). Based on MDBA modelling, ‘Low Security’ entitlements are expected to yield 0.5/ML, thus, twice the amount of entitlements are required to achieve target yields. The CEWH register indicates that, of the target of 2,075,000 ML, ‘High Security’ entitlements are expected to yield 837,353 ML, while ‘Low Security’ entitlements are expected to yield only 623,977 ML, leaving a shortfall of 161,640 ML, or ~161 GL. Based on the modelling parameters, an additional 322 GL of ‘Low Security’ holdings would be required to yield this amount and meet the target of 2075 GL. Thus, currently, the CEWH portfolio is only partly ‘hedged’ against the risk associated with ‘Low Security’ entitlements. However, the process of water recovery for environmental purposes is ongoing, and residual short-fall may also be accounted for by entitlements held by state authorities rather than the CEWH. Therefore, it is possible that if future climatic conditions are similar to the past 15 years that the target long-term annual average of 2075 GL may be available for environmental management.

In the context of climate change predictions, we suggest there are substantial risks associated with the water entitlements portfolio held by the CEWH that may threaten the availability of environmental water. Similar risks may apply in other regions impacted by climate change, such as California where ‘junior’, low reliability water rights make up a large proportion of the rights established for environmental purposes (Kendy et al. 2018). In the lead-up to the GFC, preference for high-risk ‘sub-prime’ products in the finance sector reflected an underlying cognitive bias and belief that the mortgage market was infallible (Ülgen 2013); even during a recession enough people would continue to prioritise repayments, and bonds would continue to yield profits. A similar ethos underlies current approaches to delivering the Basin Plan; purchasing a large number of low-yielding water entitlements should generate a reliable amount of water over the long term. In both cases, confidence in the efficacy of the approach rests on the assumption that the future behaviour of complex systems will be similar to that of a defined period in the past. Thus, people will pay mortgages and, the next 100 years of rainfall, river flow and dam storage will reflect the past 100 years.

In some respects, problems related to modelling LTAAY, and assumptions about future water availability, are characteristic of efforts to operationalise adaptive governance to manage complex social-ecological systems involving nonlinear environmental conditions and uncertainty (Chaffin and Gunderson 2016; Folke et al. 2005). Adaptive governance aims to achieve resilience, whereby governance arrangements are capable of responding to unpredictable change, rather than striving to prevent or control the trajectory of social-ecological systems (Folke 2006). In the context of climate change, Boltz et al. (2019) argue that water is the ‘master variable’; water availability, and the capacity to implement genuine adaptive management of water resources, will determine the resilience of wider social-environmental systems. The MDB is a complex system which represents many of the large-scale challenges of adaptive governance in river basins globally (Bischoff-Mattson and Lynch 2017). There is considerable uncertainty about the impact of climate change on future water availability in the MDB, and the allocation of water to entitlements. This uncertainty constrains the ability of policy makers to implement adaptive governance in the MDB, and for environmental management more generally (Rijke et al. 2012). However, in the case of the Basin Plan, LTAAY was knowingly modelled by intentionally excluding best available climate change predictions. This suggests that political decision making is underlain by cognitive bias; the implicit assumption that complex social, ecological and economic systems will remain stable, and that future conditions will mirror the past.

If future climatic conditions are more similar to recent decades compared with long-term trends, our estimates based on allocations to ‘Low Security’ entitlements over the last 15 years suggest that the portfolio is likely to yield as little as 1565.4 GL, which is a shortfall of 553 GL. Thus, in the context of climate change, the amount of available environmental water may fall below the target when rainfall, runoff and dam storage decline, resulting in reduced allocation of water to ‘Low Security’ entitlements, and lower than expected amounts of water available for environmental purposes. The Basin Plan offers a powerful framework for addressing over-allocation and redistributing scarce resources to improve the condition of degraded ecosystems and environmental assets. We believe these ambitions can be realised by adjusting the CEWH entitlements portfolio, and improving the reliability of environmental water.

### Improving the Reliability of Environmental Water

We are concerned that yields from ‘sub-prime’ or ‘Low Security’ water entitlements will remain low, or decline in future years. We have two recommendations for improving the reliability of environmental water. The first is for the MDBA to re-model LTAAY using the best available science about future climate scenarios and possible impacts on surface water availability in the MDB, such as the IPCC’s Fifth Assessment Report (IPCC 2014). The MDB is recognised as a likely hot-spot for water security vulnerability by 2050, under a medium emissions scenario. Modelling of the amount of ‘Low Security’ entitlements required to yield an equivalent amount of ‘High Security’ entitlements should be based on predicted near-term and long-term risks to water resources associated with warming and drying. It is likely that recalculating LTAAY to include future climate change predictions would probably lower the expected reliability of ‘Low Security’ entitlements, and necessitate an expansion of the CEWH portfolio.

Our second recommendation is to review how climate change will impact different water security products using the historic allocation data presented in this paper, and, on this basis, to hedge more effectively against any risk in changes to reliability. If climate and rainfall remain similar to the past 100 years and ‘Low Security’ entitlements yield 0.5/ML, the target annual average amount of 2075.4 GL could be achieved by either hedging ‘Low Security’ entitlements in their entirety by purchasing an additional 322 GL worth of these entitlements, or by purchasing an additional 161 GL of ‘High Security’ entitlements.

However, climate projections suggest that future yields will reduce. There has to date been no formal analysis of how different water products (‘High’ and ‘Low’ security) will fair under different climate change scenarios. In different river basins, the water sharing arrangements will have differing implications for future security of these products (Horne 2017). To further complicate things, the above cap water, which is currently providing environmental benefits and was considered in setting the water recovery target for the environment, may take a larger hit from climate change than any water entitlements. Furthermore, recent observations about catchment rainfall–runoff relationship changes during the Millennium Drought suggest future dry periods may produce significantly less runoff than previously expected (Saft et al. 2015, 2016). The Basin Plan process is adaptive allowing reviews at regular intervals. We recommend that the next Basin Plan review incorporate an assessment of the security of supply of different water products under climate change, and that this be done using stochastic data to allow a probabilistic assessment of yield.

As a very preliminary assessment, future climate and rainfall could be considered to be similar to the past 15 years and the Millennium Drought, during which time ‘Low Security’ entitlements yield between 0 and 0.4, and average of 0.2/ML. If this were the case, the shortfall between the target of 2075.4 GL and actual yields from ‘Low Security’ entitlements may be up to 511 GL. Given that ‘Low Security’ entitlements are likely to yield only 0.2/ML, an additional 2755 GL worth of these entitlements, or 511 GL of ‘High Security’ entitlements would be required to make up the short-fall.

Figure 2 indicates that if a larger number of ‘Low Security’ entitlements are purchased to hedge the existing portfolio, it may also be possible to reduce the amount of entitlements required by acquiring these entitlements from river basins in Queensland, where these entitlements are typically higher yielding (0.6/ML) compared with Victoria (0.2/ML) and NSW (0.2/ML). However, the MDB recovery target includes sub-basin targets, which may limit substitution between states. Importantly, our analysis demonstrates that, in contrast to Victoria and NSW, there was a significant difference between allocations to ‘Low Security’ entitlements in Queensland during and after the Millennium Drought. Allocations to ‘Low Security’ entitlements remained considerably higher than equivalent allocations in other states of the MDB. However, we have not investigated the socio-political consequences of purchasing a large number of entitlements from a single basin or region, and would advise caution to avoid negative consequences for farming communities. Furthermore, there is a need to access all types of allocations across all sub-catchments as our analysis (Table 2) is limited to ‘Low Security’ entitlements (as opposed to other entitlements, such as ‘Bulk’ entitlements), and limited to entitlements held for the purpose of environmental watering.

The options we present to improve the reliability of environmental water, by either re-model LTAAY or by using historic allocation data to estimate shortfall, both involve revisiting the CEWH portfolio. Both options could be achieve by purchasing a combination of ‘Low Security’ and ‘High Security’ assets, depending on how the expected yield of ‘Low Security’ entitlements is calculated.

## Conclusion

Securing water for environmental purposes in over-allocated river basins is a global challenge. Water markets offer a mechanism for reallocating resources, however, to be effective, environmental water rights must be reliable. In Australia, Chilé (e.g., Kretschmer et al. 2017), America (e.g., Kendy et al. 2018) and elsewhere (e.g., Escriva-Bou et al. 2020), environmental water rights are typically less reliable than agricultural water rights. Our findings indicate that the CEWH is probably over-reliant on ‘Low Security’ entitlements to deliver environmental flows in the MDB. We recommend adopting methods established by the finance sector to reduce the risk associated with low-yielding products, including ‘hedging’ ‘Low Security’ entitlements. This could be achieved by purchasing an additional 322–2755 GL of ‘Low Security’ entitlements, or an additional 160–511 GL of ‘High Security’ entitlements, depending on future climate scenarios. Other regions that are likely to be similarly impacted by climate change, such as California, may face similar challenges associated with the declining reliability of ‘Low Security’ water entitlements (known as ‘junior’ rights in America) in the future.

The commodification of natural resources, including water, is one of the most lucrative investment frontiers of the 21st century. The challenges facing the implementation of the Basin Plan, and ‘Low Security’ water entitlements, are highly characteristic of what Sullivan (2013) refers to as the ‘financialization of environmental conservation’. There are both strengths and weaknesses of using economic approaches to manage scarce natural resources, however, some approaches involve greater risks than others. For example, while carbon offsetting is an imperfect science (Bumpus & Liverman, 2008), the link between offset licences and reducing carbon emissions is transparent. In contrast, the link between financial products and natural resources that encompass the Australian water market is much less transparent. Characteristics, including the variable reliability of products, and the uncertainty around the yields of those products, emulate Wall Street finance in the lead up to the GFC. In particular, there are similar risks associated with using ‘Low Security’ water entitlements for the Basin Plan, and underwriting CDOs with sub-prime mortgages. In both cases, the expected yield of high-risk products was calculated on the premise that future conditions will be similar to average conditions in the past.

In the context of climate change, it is possible that future decades in Australia’s MDB will be considerably dryer than the past 100 years (Dey et al. 2019). If this scenario eventuates, ‘Low Security’ entitlements may yield significantly less water than models anticipate. This outcome could also affect the ability of irrigators who rely on ‘Low Security’ entitlements to plan for the coming agricultural season (Khan et al. 2010). In his recent report to the Governor of South Australia, Commissioner Bret Walker (2019) recounted two opposing impressions from his experience of conducting the Royal Commission:

‘The first. is of admiring praise for the enactment of the Water Act 2007….The second is one of deep pessimism whether the objects and the purposes of the Act and the (Basin) Plan will be realised’. (p.11).

We suggest that it is entirely possible to achieve the objectives of the Basin Plan by adjusting the water entitlement portfolio held by the CEWH. This would involve remodelling LTAAY, further hedging of ‘Low Security’ entitlements, or purchasing additional ‘High Security’ entitlements, or a combination of these options. To date, the issue of entitlement security has featured very little in debates about the implementation of the Basin Plan. A significant opportunity exists in the next Basin Plan review to explicitly consider the implications of climate change on the security of supply for different water products. This would help to inform and improve the water recovery approach and the reliability of the CEWH portfolio, and restore faith in the capacity of the Basin Plan to achieve environmental outcomes.

The evolution of market-based approaches for managing natural resources, including the distribution of water, reducing carbon emissions and preventing the net loss of wetlands, is an international phenomenon. However, market principles are not always directly transferrable to natural landscapes, and present challenges for ecological restoration. Many of these challenges are similar to those experienced in the finance sector. As such, finance economics may also offer avenues for overcoming challenges, and achieving the objectives of environmental policy. Water management in river basins is often framed as a tug-of-war between two adversaries: farmers and the environment. In reality, mismanagement will have negative implications for both. A more useful juxtaposition is between current compared with future goals and priorities. In the case of the MDB, without adjustment, the existing environmental water portfolio may result in the need for more drastic measures in the future, such as reduced allocations to both environmental and irrigation entitlements.

The Australian example, and our suggestions for improving the security of environmental water in the MDB, also offer insight about preventing similar situations from arising in other regions. For example, in the case of California, it may be necessary to ‘hedge’ low-yielding ‘junior’ rights with more secure ‘senior’ rights. The climate is changing. Now is the time to increase the reliability of environmental water and, in doing so, safeguard future allocations for farming communities and ecological recovery.

## Footnotes

1 We acknowledge that this assumption has been widely critiqued. However, the link between the financial product and the environmental asset is considerably more transparent than other examples of offsetting.

2 Now the Commonwealth Environmental Water Office.

3 Entitlements held by the CEWH under the Basin Plan do not include any ‘Low Security’ entitlements in South Australia. Similarly, the CEWH portfolio does not include entitlements in the Australian Capital Territory.

4 Examining the yield of ‘other’ entitlements, including conveyance entitlements, is beyond the scope of this paper. However, we suggest research is needed to explore the yields, and the implications for environmental watering.

5 Historic allocation data is available as supplementary material.

6 All seasonal allocations and determinations for the state of Queensland were provided by the Sunwater Water Accounting Department in personal email communication.

7 Entitlements from South Australia are ‘High Security’ only, and thus data from South Australia are not included in this research.
